# Factors associated with time to disability progression in patients with multiple sclerosis

**DOI:** 10.17843/rpmesp.2022.394.11433

**Published:** 2022-12-26

**Authors:** Anibal Arteaga-Noriega, John Fredy Castro-Álvarez, Dione Benjumea-Bedoya, Johanna Gutiérrez-Vargas, Angela Segura-Cardona, Difariney González-Gómez, José Zapata-Berruecos

**Affiliations:** 1 Epidemiology and Biostatistics Research Group, Universidad CES, Medellín, Colombia. Universidad CES Epidemiology and Biostatistics Research Group Universidad CES Medellín Colombia; 2 Family and Community Health Research Group, Remington University Corporation, Medellín, Colombia. Remington University Corporation Family and Community Health Research Group Remington University Corporation Medellín Colombia; 3 Neurosciences and Aging Research Group, Remington University Corporation, Medellín, Colombia. Remington University Corporation Neurosciences and Aging Research Group Remington University Corporation Medellín Colombia; 4 National School of Public Health, University of Antioquia, Medellín, Colombia. University of Antioquia National School of Public Health University of Antioquia Medellín Colombia; 5 Neurological Institute of Colombia, Medellín, Colombia. Neurological Institute of Colombia Medellín Colombia

**Keywords:** multiple sclerosis, Multivariate Analysis, disability evaluations

## Abstract

**Objectives.:**

To determine the sociodemographic, clinical and radiological factors associated with time to disability progression in patients with multiple sclerosis (MS).

**Materials and methods.:**

Cross-sectional descriptive study with an analytical component, based on clinical records of patients at the Neurological Institute of Colombia, between 2013 and 2021. Progression to disability in MS patients was defined as the time to an increase of at least 0.5 points in the EDSS (Expanded Disability Status Scale) score, sustained for at least six months. A Cox regression model was used to estimate the survival function and Hazard Ratios (HR) with their 95% confidence intervals (95% CI).

**Results.:**

We included 216 patients, of whom 25% progressed to disability, median survival was 78 months (95% CI: 70-83), active lesions (HR = 1.94; 95% CI: 1.10-3.44), cerebellar complications (HR = 2.03; 95% CI: 0. 99-4.16), being male (HR = 2.5; 95% CI: 1.32-4.73), and having neurological diseases (HR = 2.18; 95% CI: 1.03-4.61) were associated as risk factors. While relapsing remitting MS (HR = 0.63; 95% CI: 0.31-1.26) and age at diagnosis less than 40 years (HR = 0.96; 95% CI: 0.53-1.76) were associated as protective factors**.**

**Conclusions.:**

Progression is affected by many factors, and there is no single independent factor.

## INTRODUCTION

Approximately 2.8 million people worldwide are affected by multiple sclerosis (MS), a chronic inflammatory and neurodegenerative disease of the brain and spinal cord, that typically causes severe physical disability and has a higher prevalence in women ^1^. MS generates a great personal and socioeconomic burden; the average age of onset of the disease is 30 years and after approximately 25 years of diagnosis, most patients will need help to walk ^2^. The presentation of this disease is diverse and may include sensory and vision problems, motor deficits, fatigue, pain, and cognitive deficits. The variation in clinical manifestations correlates with the occurrence and expansion of lesion sites in the central nervous system ^(^^3^. Lesions are caused by infiltration of immune cells across the blood-brain barrier which promotes inflammation, myelin loss, gliosis and neuroaxonal degeneration ^4^.

The clinical manifestations are the result of motor disturbances of the sensory, visual, and autonomic systems ^5^. The most representative clinical features of the disease are optic neuritis, myelitis, brain stem/brain symptoms, cerebellum, and paroxysmal events ^6^. The McDonald criteria are the most widely used diagnostic criteria; they consider the clinical characteristics of the disease, the lesions and diagnostic aids that demonstrate the diffusion of the lesions in the area (different regions) and in time (evolution of the disease); as well as the intrathecal presence of immunoglobulins (Ig) ^7^.

The Expanded Disability Status Scale (EDSS) is the most widely used scale for monitoring the disease, and has the advantage of being able to be used during routine neurological examinations ^8^. Disability in MS patients is assessed by the EDSS on a range of 0 to 10, where 0 indicates no disability and 10 indicates death from MS ^9^. The scale allows the monitoring of disability progression, defined in clinical trials as the increase of the EDSS value by at least 0.5 points, remaining constant for at least six months, in the absence of flare-ups ^10^. The problems caused by the disease are associated with cognitive and physical alterations that can lead the patient to a deterioration in quality of life ^11^. The factors that influence the progression of disability are all the characteristics that inform about the evolution of the disease and that can guide how it will evolve over time ^12^.

Disability is one of the most important aspects to evaluate regarding MS, for this reason, most studies relate prognostic factors to the EDSS score ^13^. Some good and bad prognostic factors for the progression of disability in MS have been described in the literature, which have helped to identify the risk in patients ^2^. However, the factors described so far have only been useful up to a given level of disability, but a statistical model that integrates sociodemographic, clinical, and radiological variables and explains the time of disability progression in MS in Colombia has not been obtained. Therefore, the aim of this study was to determine the sociodemographic, clinical, and radiological factors associated with disability progression in MS patients.

**KEY MESSAGES**

Motivation for the study: multiple sclerosis (MS) is a complex disease that requires management by different disciplines. Data on Latin American patients is scarce, therefore, the usually used theoretical references are from other population groups.

Main findings: sociodemographic (male), clinical (concomitant neurological diseases) and radiological (active lesions in magnetic resonance imaging) factors were found to be associated with disease progression.

Implications: taking the above into account when approaching patients in daily clinical practice, it is possible to identify when their condition has greater possibilities of progression and thus eventually prevent complications.

## MATERIALS AND METHODS

### Study design and context

We conducted an analytical observational follow-up study of an open retrospective cohort (patients entered and left) that included information from the medical records of patients who consulted INDEC (Neurological Institute of Colombia) between 2013 and 2021. INDEC, located in the city of Medellin, is a referral center for the control of the disease, where care is provided ranging from outpatient consultation to intensive care unit.

### Participants

The medical records of 216 patients were included by a census of the period. We included patients who met the inclusion criteria: participants with confirmed diagnosis of MS according to the McDonald criteria ^14^^)^ evaluated by a neurologist during the control appointments reported in the medical record as follow-up; those who had visited INDEC between 2013 to 2021; participants that, at the time of control appointments, were of legal age; those who had EDSS score and were residents of the metropolitan area of Valle de Aburrá. Patients were electronically identified in the institution’s database according to diagnostic code G35 (ICD 10 classification) ^15^; then the criteria were verified manually.

### Variables

The time to disability progression was the outcome variable, defined according to previous studies, as an increase in the EDSS scale by at least 0.5 points, sustained for at least six months^10^^,^^16^. The patient who progressed to disability was the one who presented such an increase during the study period. Demographic variables such as sex, cohabitation status and age were considered as possible associated factors. Clinical variables were also considered such as the disease phenotype, age at diagnosis (younger and older than 40 years), initial symptoms by EDSS functional system, complications by functional system, disease-modifying treatment, comorbidities (mental disorders such as depression, bipolarity, neurological diseases such as epilepsy, stroke) and their treatment (antidepressants, cardiovascular). Areas with demyelinating lesions on MRI were considered as radiological variables. The time elapsed from symptom onset to diagnosis was also included.

### Sources of information and bias

A database was compiled with information from the medical records. The minimum number of EDSS assessments was two. Typed information was verified and all the information collected was subjected to quality control. The EDSS assessments were confirmed with a neurologist physician, member of the research group. Some patients were lost during follow-up and the last patient data collection was carried out in August 2021. The patients who, for some reason, did not complete follow-up were considered as censored data, this information was included and analyzed until the last time they participated in the study.

### Statistical analysis

An exploratory analysis of the data was conducted in order to detect any atypical behavior. We used absolute and relative frequencies for qualitative variables during the univariate analysis. For quantitative variables, distribution was determined using the Shapiro Wilk test. Since the data were not normally distributed, we used medians and interquartile ranges (IQR). Differences between patients who progressed to disability were assessed with the chi-square or Mann Whitney U test, as appropriate.

Survival time was expressed as median and IQR. We created curves showing the changes and the calculation of survival probabilities over time by using the Kaplan-Meier (K-M) method; thus, each period represented a time point. We used the total number of patients exposed in that period as the divisor to calculate the risk at the time of each event (progression). Survival (time to event) was compared for each of the covariates in order to identify factors associated with the outcome. We used the Log-Rank test (H0 = curves cross at some point, H1 = curves do not cross) (p < 0.25), applied clinical judgment and also reviewed literature.

Factors associated with the progression of disability were identified by simple Cox regressions for the variables that were found to be statistically significant during the bivariate analysis, considering the Hosmer Lemeshow criterion (p ≤ 0.25), and calculating coefficients, statistical significance, Hazard Ratio (HR) and adjusted Hazard Ratio (aHR) with their respective 95% confidence intervals (95% CI) and Akaike’s information coefficient (AIC) and clinical significance. Statistical significance was defined as a p-value < 0.05. From these regressions, we identified the variables to be included in the multivariate Cox regression model, which were included from lowest to highest AIC. Proportional hazards assumptions were evaluated for the variables that entered the final model (H0 = proportional hazards assumption is met, H1 = proportional hazards assumption is not met). Patients who did not progress to disability also contributed to the model estimation. Analyses were carried out with Stata version 17 (College Station, TX).

### Ethical aspects

The ethics committee of the CES university approved this research (Act 148 of 2020), qualifying it as a minimum risk study according to resolution 8430 of 1993 of the Colombian Ministry of Health.

## RESULTS

### Sociodemographic, clinical, and radiological characterization

**Figure 1:**
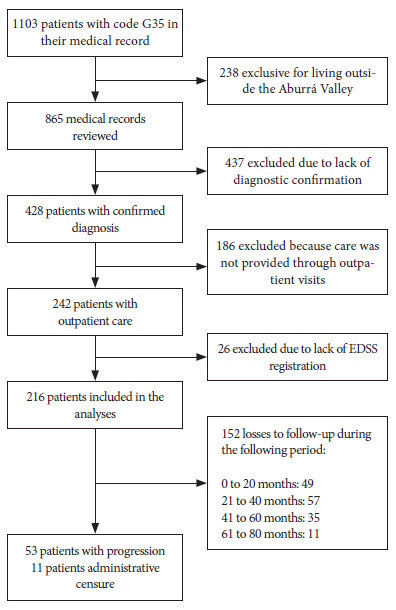
Flowchart of the inclusion of patients with multiple sclerosis for disability progression analysis.

A total of 216 patients were included in the study (Figure 1). We present the distribution of patients who had progression of disability (PD) and those who did not have progression of disability (NP). Twenty-five percent of participants showed an increase in the EDSS score, which was defined as the increase of at least 0.5 points sustained for at least six months between assessments. The median age at the beginning of follow-up was 47 years (IQR: 38-56) for the PD group, and 41 years (IQR: 31-53) for the NP group. Furthermore, 56.6% of those in the PD group and 71.2% of those in the NP group were diagnosed before the age of 40 years. Most participants were women; 65.1% in the PD group and 80.9% in the NP group. The relapsing-remitting phenotype (RR) was the most frequent among participants, with 52.8% in the PD group and 17.8% in the NP group. On the other hand, 30% of participants from the PD group were found to have the primary progressive phenotype (PP), meanwhile, the secondary progressive phenotype (SP) was found in 17% of the participants.

The median time from the onset of symptoms to diagnosis was the same for participants from both groups; the median number of years to diagnosis was higher in the PD group at 8.16 years (IQR: 3.75-12.58). Cardiovascular drugs (antihypertensives and antiarrhythmics) were used in 24.5% of the participants who had PD. The consumption of antidepressants was higher in those with PD (37.7%), whereas 13.2% of participants from the PD group had mental illness and 16.9% had other neurological diseases. Regarding complications, 64.1% of those in the PD group had cerebellar type complications, 15.1% had bladder and bowel complications and 52.8% had vision problems. Sensory onset symptoms were found in 36.2% of those in the NP group, while 30.2% had cerebellar disease onset symptoms. Among those in the PD group, 82.7% had spinal cord lesions identified by MRI and 40.4% had active lesions (Table 1).

**Table 1:** Characteristics of patients with multiple sclerosis according to progression of disability.

Variable	Progression to disability (PD)		Non-progression to disability (NP)		p-value ^b^	p-value ^c^
	n=53	%	n=163	%		
Age ^a^	47	38−56	41	31−53	0.010	0.010
Sex						
Men	19	35.9	31	19.0	0.010	0.010
Women	34	64.1	132	81.0		
Age at diagnostic						
<40	30	56.6	116	71.2	0.070	0.010
>40	23	43.4	47	28.8		
RR phenotype						
Yes	28	52.8	29	17.8	0.010	0.010
No	25	47.2	134	82.2		
PP phenotype						
Yes	16	30.2	15	9.2	0.010	0.010
No	37	69.8	148	90.8		
SP phenotype						
Yes	9	17.0	14	8.6	0.140	0.160
No	44	83.0	149	91.4		
Time of symptoms to diagnosis (months) ^a^	12	8−48	12	6−36	0.930	0.890
Years since diagnosis ^a^	8,16	3.75−12.58	6,1	6−36	0.010	0.010
Cardiovascular medications						
Yes	13	24.5	21	12.9	0.070	0.080
No	40	75.5	142	87.1		
Antidepressants						
Yes	20	37.7	31	19.0	0.050	0.060
No	33	62.3	132	81.0		
Mental disorders						
Yes	7	13.2	12	7.4	0.100	0.190
No	46	86.8	151	92.6		
Neurological Diseases						
Yes	9	17.0	12	7.4	0.030	0.040
No	44	83.0	151	92.6		
Cerebellar complications						
Yes	34	64.2	60	36.8	0.010	0.010
No	19	35.8	103	63.2		
Vision problems						
Yes	28	52.8	72	44.2	0.340	0.160
No	25	47.2	91	55.8		
Bowel and bladder complications						
Yes	8	15.1	19	11.7	0.670	0.540
No	45	84.9	144	88.3		
Sensitivity symptoms						
Yes	11	20.7	59	36.2	0.030	0.080
No	42	79.3	104	63.8		
Brainstem symptoms						
Yes	2	3.8	1	0.6	0.040	0.010
No	51	96.2	162	99.4		
Cerebellar symptoms						
Yes	16	30.2	29	17.8	0.020	0.030
No	37	69.8	134	82.2		
Spinal cord injuries						
Yes	43	82.7	45	27.9	0.120	0.160
No	9	17.3	116	72.1		
Active injuries						
Yes	21	40.4	44	27.3	0.010	0.030
No	31	59.6	117	72.7		

a Median and interquartile range; ^b^ Chi-square test or Mann Whitney U test; ^c^ Log-Rank test.

RR: relapsing remitting. PP: primary progressive.

### Overall survival

**Figure 2:**
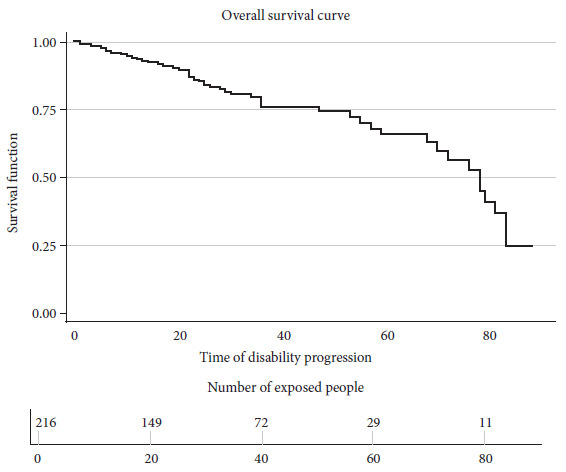
Curve for disability progression of the 216 patients who were included in the study, showing the time to disability progression exposing failures (progression). n: number

The overall median survival time was 78 months with an interquartile range of 70-83 months (Figure 2). The cumulative follow-up time was 70-60.5 months. More events were reported during months 22 and 36, with four in each month. The period with the most patient losses was month 32, with seven losses.

### Associated factors

The Log-Rank test was used for the bivariate analysis, after which we included the candidate variables in the simple models according to the p-value and clinical significance, in order to subsequently perform the adjusted Cox regression model. Active injuries (aHR= 1.94; 95% CI 1.10-3.44), being male (aHR= 2.5; 95% CI 1.32-4.73) and having neurological diseases (aHR= 2.18; 95% CI 1.03-4.61) were associated with disability progression. On the other hand, having had cerebellar complications (aHR = 2.03; 95% CI 0.99-4.16), RR phenotype (aHR= 0.63; 95% CI 0.31-1.26) and age at diagnosis of less than 40 years (aHR = 0.96; 95% CI 0.53-1.73) were factors not associated with disability progression (Table 2).

**Table 2:** Simple and adjusted models of disability progression in patients with multiple sclerosis.

Variable	Simple Cox regression			Cox multivariate regression			
	HR	95% CI	p-value	aHR	95% CI	p-value	p-value ^g^
Presence of active lesions ^a^	1.82	3.18-450.23	0.034	1.94	1.10-3.44	0.022	0.759
Having presented cerebellar complications ^b^	2.68	1.53-4.71	0.001	2.03	0.99-4.16	0.053	0.374
Male ^c^	3.03	1.66-5.52	0.001	2.50	1.32-4.73	0.005	0.995
Having RR phenotype ^d^	0.38	0.22-0.66	0.001	0.63	0.31-1.26	0.196	0.885
Having other neurological diseases ^e^	1.75	0.93-4.62	0.073	2.18	1.03-4.61	0.040	0.990
Age at diagnosis less than 40 years ^f^	1.48	0.86-3.60	0.153	0.96	0.53-1.73	0.910	0.460

Reference categories: ^a^ no active lesions; ^b^ no cerebellar complications; ^c^ female; ^d^ no RR phenotype; ^e^ no neurological disease; ^f^ age at diagnosis greater than 40 years; ^g^ Cox proportional hazards assumption test.

HR: hazard ratio; ^a^HR: adjusted hazard ratio; 95% CI: 95% confidence interval.

## DISCUSSION

We found that male patients had a 2.5 faster progression than female patients. This result is supported by studies showing that women are less likely to progress to disability or do so more slowly, although the disease is more frequent in women ^17^. Ribbons *et al*. analyzed data from 15,826 MS patients from 25 countries comparing EDSS scores by sex, and male MS patients showed a faster EDSS progression ^18^.

On the other hand, MRI is considered a useful tool for the identification of demyelinating areas, lesions, and blood-brain barrier rupture ^19^. We found that the patients with active lesions on MRI showed faster progression. Studies have reported that the importance of finding active lesions lies in the opportunity to predict the clinical deterioration of the patient and therefore the loss of brain volume; consequences in the patient will depend on the site of the lesion ^20^. Harrison *et al*. reported that, using magnetic resonance imaging, cortical lesion burden, including the number and volume of lesions, was closely related to physical disability and cognitive dysfunction ^21^. On 2018, a study by Eshaghi *et al*., that included 1417 subjects (253 with clinically isolated syndrome, 708 with relapsing-remitting multiple sclerosis, 128 with secondary progressive multiple sclerosis, 125 with primary progressive multiple sclerosis and 203 healthy control subjects); reported that, the first regions to atrophy in patients with clinically isolated syndrome and RR multiple sclerosis were the posterior cingulate cortex and precuneus, followed by the medial cingulate cortex, brainstem and thalamus. A similar atrophy sequence was detected in PP multiple sclerosis with involvement of the thalamus, cuneus, precuneus and pallidum, followed by the brainstem and posterior cingulate cortex. The cerebellum, caudate and putamen showed early atrophy in RR phenotype cases and late atrophy in PP phenotype cases ^22^.

Our results show that 64.1% of the patients who progressed to disability had cerebellar lesions. Such complications have been related to increased scores on the disability scale and have even played an important role in the progression from isolated clinical syndrome to MS^23^. In MS patients, demyelination of the gray matter of the cerebellum, more than any other brain region, is five times greater than the demyelination of the white matter ^24^. This may be a consequence of overlying meningeal inflammation in the deep folia, which harbors a static inflammatory milieu (such as cytokines and immunoglobulins) ^25^. Therefore, overlying inflammation in the cerebellum may amplify other pathological mechanisms such as retrograde neurodegeneration secondary to white matter lesions ^24^^,^^25^. Early cerebellar presentation is associated with unfavorable outcomes, whereas early brainstem presentation is associated with a favorable prognosis ^26^. These presentations can be used as prognostic markers of MS and guide the therapeutic approach.

We found that having a history of neurological diseases was associated with progression, this has been previously reported by studies that found that this type of patients is more prone to progression. Epilepsy seems particularly related to an increased risk of physical disability in patients with relapses and remissions ^27^. The prevalence of psychiatric illness in the systematic review by Marrie *et al*. was quite high (i.e., 23.7% for depression, 21.9% for anxiety and 5.83% for bipolar disorder) ^28^.

The simple model we used during the analysis showed that age at diagnosis influenced disability in MS patients. However, multivariate regression revealed that it did not influence MS disability. In a 2011 study, Scalfari *et al*. reported that age was an independent and important factor contributing to disease progression. These authors report that the age at onset of RR phenotype disease and advancing age (current age) affect the accumulation of disability, regardless of disease duration, largely by increasing the likelihood of experiencing a progressive course and shortening the latency to progression ^29^. In addition, the authors also suggest that age stratification, which has been little used so far, may be advantageous, especially if the primary outcome is the occurrence of secondary progressive multiple sclerosis^29^. Early stages of the disease, especially during younger ages, represent a window of opportunity for future treatments that should focus on preventing or delaying the onset of secondary progression, the main determinant of the development of permanent disability.

Our results show that, for both, the simple regression model and the multivariate regression model, the RR phenotype was not a factor influencing MS disability progression. Stewart *et al*. conducted a prospective follow-up study on 136,462 patients with RR phenotype and found that a higher recurrence rate was related to a higher disability accumulation ^30^. Studies suggest that the PP phenotype portends a worse prognosis than the RR phenotype or SP phenotype in terms of disability. However, progressive disease and the rate of post-progression disability accumulation appear to be age-dependent and do not correlate with the rate of pre-progression disability accumulation ^29^. The previously mentioned studies that reported that patients with PP phenotype are more likely to progress in disability support our results, which show that more than 30% of patients had this phenotype.

Since this was a follow-up cohort study, some participants were lost during follow-up. In order to control for this type of bias, we considered that each patient contributed time while on follow-up, ensuring that we obtained the most accurate information. The analysis included the follow-up of 167 people for at least 20 months, which was equivalent to 77.3% of the study population. However, at 40 months about 50% of the population had been lost, which limits the identification of the time of disability progression in this group of participants. Additionally, we used non-probabilistic sampling, therefore, the results cannot be generalized to the department of Antioquia. Patients were not at the same stage of the disease when they entered the study, therefore, they had different levels of disability; in addition, there were cases lost during follow-up, although the data from the follow-up period was available in the medical records. However, this process was carried out in the framework of a clinical interview with a neurologist, who helped reviewing all reported consultations.

In conclusion, disability progression in MS patients is affected by several factors, with no single independent factor. The median time to progression was 72 months. Active lesions found on MRI and male sex were associated with greater disability progression, with statistically significant results in the multivariate model. Therefore, the correct and timely understanding of the risk factors associated with disability progression can help both to counsel patients and to improve the approach and provide evidence-based treatment recommendations, which helps to improve the prognosis and quality of life of patients. 
