# Supplementary material for: Factors associated with time to disability progression in patients with multiple sclerosis
Source: Rev Peru Med Exp Salud Publica. 2022 Dec 26;39(4):442–9. doi: 10.17843/rpmesp.2022.394.11433 (PMC11397597; doi:10.17843/rpmesp.2022.394.11433)
Supplement: Translation

# Translation: Factores asociados con el tiempo de progresión de la discapacidad en pacientes con esclerosis múltiple

**Authors:** Anibal Arteaga-Noriega, John Fredy Castro-Álvarez, Dione Benjumea-Bedoya, Johanna Gutiérrez-Vargas, Angela Segura-Cardona, Difariney González-Gómez, José Zapata-Berruecos

**Affiliations:** 1 Grupo de Investigación en Epidemiología y Bioestadística, Universidad CES, Medellín, Colombia; 2 Grupo de Investigación en Salud Familiar y Comunitaria, Corporación Universitaria Remington, Medellín, Colombia; 3 Grupo de Investigación en Neurociencias y Envejecimiento, Corporación Universitaria Remington, Medellín, Colombia; 4 Facultad Nacional de Salud Pública, Universidad de Antioquia, Medellín, Colombia; 5 Instituto Neurológico de Colombia, Medellín, Colombia

**Keywords:** Esclerosis Múltiple, Análisis Multivariante, Evaluación de la Discapacidad

## Abstract

**Objetivos.:**

Determinar los factores sociodemográficos, clínicos y radiológicos asociados al tiempo de progresión de discapacidad en pacientes con esclerosis múltiple (EM).

**Materiales y métodos.:**

Estudio transversal analítico, basado en registros de la historia clínica de pacientes del Instituto Neurológico de Colombia, entre el 2013 y 2021. La progresión a discapacidad de los pacientes con EM se definió como el tiempo hasta un aumento de por lo menos 0,5 puntos en el valor de la EDSS (del inglés *Expanded Disability Status Scale*), sostenido por al menos seis meses. Se usó un modelo de regresión de Cox para estimar la función de supervivencia y los hazard ratios (HR) con sus intervalos de confianza de 95% (IC 95%).

**Resultados.:**

Se incluyeron 216 pacientes, de los cuales el 25% progresó a discapacidad, la mediana de supervivencia fue de 78 meses (RIC 95%: 70−83), las lesiones activas (HR = 1,94; IC 95%: 1,10−3,44), el sexo masculino (HR = 2,5; IC 95%: 1,32−4,73), y las enfermedades neurológicas (HR = 2,18; IC95%: 1,03−4,61) se asociaron en el modelo multivariado.

**Conclusiones.:**

La mediana de tiempo de progresión hacia la discapacidad fue de 72 meses. Las lesiones activas captadas en resonancia magnética y el sexo masculino se asociaron con mayor progresión de la discapacidad, con resultados estadísticamente significativos en el modelo multivariado.

## INTRODUCCIÓN

Aproximadamente, 2,8 millones de personas en el mundo están afectadas por la esclerosis múltiple (EM), una enfermedad crónica inflamatoria y neurodegenerativa del cerebro y la médula espinal, causando comúnmente discapacidad física grave, con mayor predominio en mujeres ^1^. La EM ocasiona una gran carga personal y socioeconómica, la edad media de aparición de la enfermedad es de 30 años y luego de, aproximadamente, 25 años del diagnóstico, gran parte de los pacientes van a necesitar ayuda para deambular ^2^. Esta enfermedad tiene una presentación heterogénea, que puede incluir alteraciones sensoriales y visuales, deficiencias motoras, fatiga, dolor y déficit cognitivo. La variación en las manifestaciones clínicas se correlaciona con la aparición y expansión de los sitios de lesión en el sistema nervioso central ^3^. Las lesiones son causadas por la infiltración de células inmunes a través de la barrera hematoencefálica que promueve la inflamación, la pérdida de la mielina, la gliosis y la degeneración neuroaxonal ^4^.

Las manifestaciones clínicas son producto de las alteraciones motoras de los sistemas sensoriales, visuales y autonómicos ^5^ ^) ^. Las características clínicas más representativas de la enfermedad son neuritis óptica, mielitis, síntomas de tallo cerebral/cerebro, cerebelo y eventos paroxísticos ^6^. Los criterios para el diagnóstico más usados son los de McDonald que consideran las características clínicas de la enfermedad, las lesiones observadas y las ayudas diagnósticas que demuestren la difusión de las lesiones en el espacio (diferentes regiones) y en el tiempo (evolución de la enfermedad); así como la presencia intratecal de inmunoglobulinas (Ig) ^7^. 

La escala expandida del estado de discapacidad (EDSS del inglés *Expanded Disability Status Scale*) es la más usada para realizar controles en la enfermedad, tiene como ventaja la forma de calificación que se puede hacer durante los exámenes neurológicos de rutina ^8^. La discapacidad en pacientes con EM es evaluada por la EDSS en un intervalo de 0 a 10, donde 0 hace referencia a ausencia de discapacidad y 10 se refiere a la muerte por EM ^9^. La escala permite la monitorización de la progresión de la discapacidad, definida en ensayos clínicos como el aumento del valor de la EDSS en al menos 0,5 puntos, que permanezcan constante durante al menos seis meses, en ausencia de brote ^10^. Los problemas que produce la enfermedad están asociados con alteraciones cognitivas y físicas que pueden llevar al paciente a un deterioro de la calidad de vida ^11^. Los factores que influyen en la progresión de la discapacidad son todas las características que informan sobre la evolución de la enfermedad y que pueden orientar cómo será su comportamiento a través del tiempo ^12^.

En la EM, uno de los aspectos más importante de evaluar es la discapacidad, por esta razón, la mayoría de estudios relacionan a los factores pronósticos con la medición de la EDSS ^13^ . En la literatura se han descrito algunos factores de buen y mal pronóstico para la progresión de la discapacidad en EM, que han ayudado a la identificación del riesgo en los pacientes ^2^. Sin embargo, los factores descritos hasta ahora solo han sido útiles hasta un nivel de discapacidad determinado, pero no se ha obtenido un modelo estadístico que integre variables sociodemográficas, clínicas y radiológicas que puedan explicar el tiempo de la progresión de la discapacidad en la EM en Colombia. Por lo tanto, el objetivo de este estudio fue determinar los factores sociodemográficos, clínicos y radiológicos asociados a la progresión de discapacidad en pacientes con EM.

**MENSAJES CLAVE**

Motivación para realizar el estudio: la esclerosis múltiple (EM) es una enfermedad compleja que requiere manejo por diferentes disciplinas, en la literatura científica existen pocos datos de pacientes latinoamericanos, por ende, los referentes teóricos son de otros grupos poblacionales.

Principales hallazgos: se encontró que factores sociodemográficos (sexo masculino), clínicos (enfermedades neurológicas concomitantes) y radiológicos (lesiones activas captadas en resonancia magnética) se asociaron con la progresión de la enfermedad.

Implicancias: teniendo en cuenta lo anterior al momento de abordar los pacientes en la práctica clínica diaria, se puede clasificar su condición con mayores posibilidades de progresión y así eventualmente prevenir complicaciones.

## MATERIALES Y MÉTODOS

### Diseño del estudio y contexto

Se realizo un estudio observacional analítico de seguimiento a una cohorte retrospectiva abierta (hubo entrada y salidas de pacientes) que incluyó información de las historias clínicas de los pacientes que consultaron al INDEC (Instituto Neurológico de Colombia) entre el 2013 al 2021. El INDEC, ubicado en la ciudad de Medellín, es un centro de referencia de control de la enfermedad, en donde se brinda atención desde consulta externa hasta unidad de cuidados intensivos.

### Participantes

Se incluyeron las historias clínicas de 216 pacientes mediante un censo del periodo, que cumplieran los criterios de inclusión: diagnóstico confirmado de EM según los criterios de McDonald ^14^ evaluados por un neurólogo en el control de la enfermedad reportado en la historia clínica en seguimiento; tener asistencia al INDEC entre el 2013 al 2021; que en el tiempo de los controles, los participantes fueran mayores de edad; tener puntaje de la EDSS en las consultas y ser residentes del área metropolitana del Valle de Aburrá. Los pacientes fueron identificados electrónicamente en la base de datos de la institución según código diagnóstico G35 (clasificación CIE 10) ^15^ y posteriormente se realizó verificación manual de los criterios.

### Variables

La variable de resultado fue el tiempo de progresión de discapacidad, definida según estudios previos, como el incremento del valor de la escala EDSS en, por lo menos, 0,5 puntos en el valor de la escala, sostenido por al menos seis meses ^10^ ^,^ ^16^. El paciente que progresó hacia la discapacidad fue aquel que presentó dicho incremento en el periodo del estudio. Como posibles factores asociados se consideraron variables demográficas como el sexo, estado de convivencia y la edad. También se consideraron variables clínicas como el fenotipo de la enfermedad, edad del diagnóstico (menor y mayor a 40 años), síntomas iniciales por sistema funcional de la EDSS, complicaciones por sistema funcional, tratamiento modificador de la enfermedad, comorbilidades (trastornos mentales como depresión, bipolaridad, enfermedades neurológicas tales como epilepsia, accidente cerebrovascular) y su tratamiento (antidepresivos, cardiovasculares). Como variables radiológicas se consideraron las áreas con lesiones desmielinizantes en la resonancia magnética. También se incluyó al tiempo transcurrido desde los síntomas de inicio hasta el diagnóstico.

### Fuentes de información y sesgo

Se elaboró una base de datos con la información de las historias clínicas. El número mínimo de valoraciones de EDSS fue de dos. Se realizó la verificación de la información digitada y el control de calidad a toda la información recopilada. Las valoraciones del EDSS fueron confirmadas con un médico neurólogo, miembro del grupo investigador. Hubo pérdidas de seguimientos en los pacientes y la última recolección de datos de los pacientes se realizó en agosto del 2021. Aquellos pacientes que, por alguna razón no completaron el seguimiento, fueron considerados como datos censurados, esta información fue incluida y analizada hasta el momento en que participaron en el estudio.

### Análisis estadístico

Se realizó un análisis exploratorio de los datos para detectar aquellos con comportamiento atípico. En el análisis univariado, para las variables cualitativas se calcularon frecuencias absolutas y relativas. Para las variables cuantitativas se determinó su distribución mediante la prueba de Shapiro Wilk. Dado que los datos no se distribuyeron de forma normal, se calcularon medianas y rangos intercuartílicos (RIC). Las diferencias entre los pacientes que progresaron hacia la discapacidad se evaluó con la prueba de chi cuadrado o U de Mann Whitney, según corresponda.

El tiempo de supervivencia se resumió con la mediana y el RIC. Con el método Kaplan−Meier (K−M) se generaron curvas que muestran los cambios y el cálculo de las probabilidades de supervivencia en el tiempo; así cada período representó un punto de tiempo. El denominador para calcular el riesgo al momento de cada evento (progresión) fue el número total de pacientes expuestos en ese periodo. Para identificar los factores asociados con el desenlace, se comparó la supervivencia (tiempo al evento) en cada una de las covariables evaluadas. Se utilizó la prueba de Log−Rank (H0 = las curvas se cruzan en algún momento, H1 = las curvas no se cruzan) (p < 0,25); además, se tuvo en cuenta el criterio clínico y revisión de la literatura.

Para identificar los factores asociados a la progresión de la discapacidad, se realizaron regresiones simples de Cox para las variables que fueron estadísticamente significativas en el análisis bivariado, se consideró el criterio de Hosmer Lemeshow (p ≤ 0,25); calculando coeficientes, significancia estadística, Hazard Ratio (HR) y Hazard Ratio ajustado (HRa) con sus respectivos intervalos de confianza al 95% (IC 95%) y el coeficiente de información de Akaike (AIC) e importancia clínica. La significación estadística se estableció con un valor de p < 0,05. A partir de estas regresiones, se identificaron las variables a incluir en el modelo multivariado de regresión de Cox; siendo ingresadas de menor a mayor AIC. Para las variables que ingresaron al modelo final se evaluaron los supuestos de riesgos proporcionales (H0 = se cumple el supuesto de riesgos proporcionales, H1 = no se cumple el supuesto de riesgos proporcionales). Los pacientes que no progresaron a discapacidad, de igual manera contribuyeron a la estimación del modelo. Los análisis se ejecutaron con el programa Stata versión 17 (College Station, TX).

### Aspectos éticos

Se contó con el aval del comité de ética de la universidad CES que aprobó la investigación (Acta 148 de 2020), calificándolo como un estudio de riesgo mínimo según la resolución 8430 de 1993 del Ministerio de Salud de Colombia.

## RESULTADOS

### Caracterización sociodemográfica, clínica y radiológica

En total fueron analizados 216 pacientes que se incluyeron en el estudio (Figura 1). Se representa la distribución según los pacientes que tuvieron progresión de la discapacidad (P) y los que no tuvieron progresión de la discapacidad (NP). El 25% de los pacientes que participaron en el estudio tuvieron aumento del valor de la EDSS tomada como el incremento de por lo menos 0,5 puntos en el valor de la escala sostenido por al menos seis meses entre valoraciones. En cuanto a la edad al inicio del seguimiento se encontró que la mediana fue de 47 años para los P (RIC: 38−56), mientras que para los NP fue de 41 años (RIC: 31−53), donde un 56,6% de los P versus 71,2% de los NP se les realizó el diagnóstico antes de los 40 años. En cuanto al sexo se encontró una mayoría de mujeres, el 65,1% en el grupo P y 80,9% en el NP del total de pacientes. La mayoría tuvieron el fenotipo remitente recurrente (RR) con un 52,8% en el grupo P y de 17,8% en el de NP, el fenotipo primario progresivo (PP) fue del 30% en el grupo P y 17% para el caso del fenotipo secundario progresivo (SP).

**Figura 1:** Flujograma de inclusión de pacientes con esclerosis múltiple para análisis de progresión de la discapacidad.

La mediana de tiempo de síntomas hasta el diagnóstico fue igual para los dos grupos, la mediana de los años de diagnóstico fue superior en los P con 8,16 años (RIC: 3,75−12,58). El 24,5% de los P tuvieron tratamiento con fármacos cardiovasculares (antihipertensivos y antiarrítmicos). El consumo de antidepresivos fue mayor en las personas P con un 37,7%, mientras que la presencia de enfermedad mental se encontró en el 13,2% de los P, el 16,9% de este grupo tiene otras enfermedades neurológicas. Respecto a las complicaciones, el 64,1% de los P han tenido complicaciones de tipo cerebeloso, 15,1% complicaciones de vejiga e intestino y el 52,8% de este mismo grupo han tenido complicaciones en la visión. Los síntomas de inicio sensitivos estuvieron presentes en el 36,2% de los NP, y el 30,2% presentaron síntomas de inicio de la enfermedad cerebelosa; el 82,7% tuvo lesiones en médula identificadas por resonancia magnética en los P y el 40,4% presentaron lesiones activas (Tabla 1). 

**Tabla 1:** Características de los pacientes con esclerosis múltiple según progresión de la discapacidad.

Variable	Progresión a discapacidad (P)		No progresión a discapacidad (NP)		Valor de p ^b^	Valor de p ^c^
	n=53	%	n=163	%		
Edad ^a^	47	38−56	41	31−53	0,010	0,010
Sexo						
Masculino	19	35,9	31	19,0	0,010	0,010
Femenino	34	64,1	132	81,0		
Edad al diagnostico						
<40	30	56,6	116	71,2	0,070	0,010
>40	23	43,4	47	28,8		
Fenotipo RR						
Sí	28	52,8	29	17,8	0,010	0,010
No	25	47,2	134	82,2		
Fenotipo PP						
Sí	16	30,2	15	9,2	0,010	0,010
No	37	69,8	148	90,8		
Fenotipo SP						
Sí	9	17,0	14	8,6	0,140	0,160
No	44	83,0	149	91,4		
Tiempo de síntomas al diagnóstico (meses) ^a^	12	8−48	12	6−36	0,930	0,890
Años desde el diagnostico ^a^	8,16	3,75−12,58	6,1	6−36	0,010	0,010
Fármacos cardiovasculares						
Sí	13	24,5	21	12,9	0,070	0,080
No	40	75,5	142	87,1		
Antidepresivos						
Sí	20	37,7	31	19,0	0,050	0,060
No	33	62,3	132	81,0		
Trastornos mentales						
Sí	7	13,2	12	7,4	0,100	0,190
No	46	86,8	151	92,6		
Enfermedades Neurológicas						
Sí	9	17,0	12	7,4	0,030	0,040
No	44	83,0	151	92,6		
Complicaciones cerebelosas						
Sí	34	64,2	60	36,8	0,010	0,010
No	19	35,8	103	63,2		
Complicaciones de la visión						
Sí	28	52,8	72	44,2	0,340	0,160
No	25	47,2	91	55,8		
Complicaciones vejiga intestino						
Sí	8	15,1	19	11,7	0,670	0,540
No	45	84,9	144	88,3		
Síntomas de sensibilidad						
Sí	11	20,7	59	36,2	0,030	0,080
No	42	79,3	104	63,8		
Síntomas tronco encéfalo						
Sí	2	3,8	1	0,6	0,040	0,010
No	51	96,2	162	99,4		
Síntomas cerebelosos						
Sí	16	30,2	29	17,8	0,020	0,030
No	37	69,8	134	82,2		
Tener lesiones en médula						
Sí	43	82,7	45	27,9	0,120	0,160
No	9	17,3	116	72,1		
Lesiones activas						
Sí	21	40,4	44	27,3	0,010	0,030
No	31	59,6	117	72,7		

a Mediana y rango intercuartílico; ^b^ Prueba de chi cuadrado o U de Mann Whitney;^ c^ Prueba de Log-Rank

RR: remitente recurrente. PP: primaria progresiva.

### Supervivencia global

La mediana global del tiempo de supervivencia fue de 78 meses con un con rango intercuartílico de 70−83 meses (Figura 2). En relación con el tiempo de observación, la sumatoria de tiempo de seguimiento fue de 7060,5 meses. En el mes 22 y 36 hubo más eventos, con cuatro en cada mes. El período donde más pérdidas de pacientes hubo fue en el mes 32 con siete pérdidas. 

**Figura 2:** Curva de supervivencia global para la presentación de progresión de la discapacidad en pacientes con esclerosis múltiple

### Factores asociados

Después de realizar el análisis bivariado por medio de la prueba de Log−Rank, se incorporaron las variables candidatas a ingresar a los modelos simples de acuerdo con el valor de p y la importancia clínica, para posteriormente realizar el modelo ajustado de regresión de Cox. Las lesiones activas (HRa= 1,94; IC 95%: 1,10−3,44), el sexo masculino (HRa = 2,5; IC 95%: 1,32−4,73) y tener enfermedades neurológicas (HRa = 2,18; IC 95% 1,03−4,61) se asociaron con la progresión de la discapacidad. Por otro lado, el haber presentado complicaciones cerebelosas (HRa = 2,03; IC 95% 0,99−4,16), el fenotipo RR (HRa= 0,63; IC 95% 0,31−1,26) y la edad al diagnóstico menor a 40 años (HRa = 0,96; IC 95% 0,53−1,73) fueron factores no asociados a la progresión de la discapacidad (Tabla 2).

**Tabla 2:** Modelos simple y ajustado de progresión de la discapacidad en pacientes con esclerosis múltiple

Variable	Regresión simple de Cox			Regresión multivariada de Cox			
	HR	IC 95%	Valor de p	HRa	IC 95%	Valor de p	Valor de p ^g^
Presencia de lesiones activas ^a^	1,82	3,18-450,23	0,034	1,94	1,10-3,44	0,022	0,759
Haber presentado complicaciones cerebelosas ^b^	2,68	1,53-4,71	0,001	2,03	0,99-4,16	0,053	0,374
Sexo masculino ^c^	3,03	1,66-5,52	0,001	2,50	1,32-4,73	0,005	0,995
Tener fenotipo RR ^d^	0,38	0,22-0,66	0,001	0,63	0,31-1,26	0,196	0,885
Tener otras enfermedades neurológicas ^e^	1,75	0,93-4,62	0,073	2,18	1,03-4,61	0,040	0,990
Edad al diagnóstico menor de 40 años ^f^	1,48	0,86-3,60	0,153	0,96	0,53-1,73	0,910	0,460

Categorías de referencia: ^a^ no tener lesiones activas; ^b^ no tener complicaciones cerebelosas; ^c^ sexo femenino; ^d ^no tener fenotipo RR; ^e^ no tener enfermedades neurológicas; ^f^ edad de diagnóstico mayor a 40 años; ^g ^Prueba de la suposición de riesgos proporcionales de Cox.

HR: hazard ratio, HRa: hazard ratio ajustado, IC 95%: intervalo de confianza al 95%

## DISCUSIÓN

Entre los principales hallazgos se encontró que los pacientes de sexo masculino tienen una progresión 2,5 más rápida que pacientes del sexo femenino. Este resultado es respaldado con estudios donde se muestra que las mujeres tienen menos posibilidades de progresión en discapacidad o lo hacen de una manera más lenta, aunque la enfermedad es más frecuente en ellas ^17^. Ribbons *et al.* analizaron datos de 15 826 pacientes con EM de 25 países comparando las puntuaciones de la escala EDSS entre sexos, y demostraron que los pacientes de sexo masculinos con EM tienen una progresión EDSS más rápida ^18^. 

Por otro lado, las imágenes de RM se consideran una herramienta útil en la identificación de zonas de desmielinización, las lesiones y ruptura de la barrera hematoencefálica ^19^. En el presente estudio se demuestra que progresan más rápido aquellos pacientes que a través de RM mostraron lesiones activas. Estudios han indicado que, la importancia de encontrar lesiones activas radica en la oportunidad de poder predecir el deterioro clínico del paciente y por ende pérdida del volumen cerebral, las consecuencias en el paciente dependerá del sitio de la lesión ^20^. Harrison *et al.* reportaron que, haciendo uso de imágenes de resonancia magnética, la carga de la lesión cortical, incluido el número y el volumen de las lesiones estaba estrechamente relacionada con la discapacidad física y la disfunción cognitiva ^21^. En un estudio realizado por Eshaghi *et al.* en el 2018 donde se incluyeron 1417 sujetos (253 con síndrome clínicamente aislado, 708 con esclerosis múltiple recurrente−remitente, 128 con esclerosis múltiple secundaria progresiva, 125 con esclerosis múltiple primaria progresiva y 203 sujetos control sanos); se encontró que, las primeras regiones en atrofiarse en pacientes con síndrome clínicamente aislado y esclerosis múltiple RR fueron la corteza cingulada posterior y el precúneo, seguidas por la corteza cingulada media, el tronco encefálico y el tálamo. Una secuencia similar de atrofia se detectó en la esclerosis múltiple PP con afectación del tálamo, cuneus, precuneus y pallidum, seguidos por el tronco encefálico y la corteza cingulada posterior. El cerebelo, el caudado y el putamen mostraron atrofia temprana en el fenotipo RR y atrofia tardía en fenotipo PP ^22^.

En el presente estudio el 64,1% de los pacientes que progresaron hacia la discapacidad presentaron lesiones cerebelosas. Dichas complicaciones han estado relacionadas con el aumento de los valores en la escala de discapacidad e incluso han jugado un papel importante en el paso de síndrome clínico aislado a EM ^23^. En pacientes con EM, más que en cualquier otra región del cerebro, se observa desmielinización en la materia gris del cerebelo, que es cinco veces más que la desmielinización de la materia blanca ^24^. Esto puede ser una consecuencia de la inflamación meníngea suprayacente en la folia profunda, que alberga un medio inflamatorio estático (como citocinas e inmunoglobulinas) ^25^. Por lo tanto, en el cerebelo, la inflamación suprayacente puede amplificar otros mecanismos patológicos como la neurodegeneración retrógrada secundaria a lesiones de la sustancia blanca ^24^ ^,^ ^25^. La presentación cerebelosa temprana se asocia con resultados desfavorables, mientras que la presentación temprana del tronco encefálico se asocia con un pronóstico favorable ^26^. Estas presentaciones pueden usarse como marcadores de pronóstico de EM y guiar el enfoque terapéutico. 

En cuanto a los antecedentes de enfermedades neurológicas se encontró que tuvieron asociación con la progresión, esto datos ya se habían reportado encontrándose que este tipo de pacientes son más propensos a la progresión. La epilepsia parece particularmente relacionada con un mayor riesgo de discapacidad física en pacientes con recaídas y remisiones ^27^. La prevalencia de enfermedades psiquiátricas en la revisión sistemática de Marrie *et al.* fue bastante alto (es decir, 23,7% para depresión, 21,9% para ansiedad y 5,83% para trastorno bipolar) ^28^

El modelo simple realizado en este estudio mostró que la edad de diagnóstico influía en la discapacidad de los pacientes con EM. Sin embargo, en la regresión multivariada se encontró que no era un factor que influía en discapacidad de la EM. Scalfari *et al.* en un estudio realizado en el 2011 redefinen la edad como un factor independiente e importante que contribuye a la evolución de la enfermedad. Estos autores reportan que la edad de inicio de en fenotipo RR y el avance de la edad (edad actual) afectan la acumulación de discapacidad, independientemente de la duración de la enfermedad, en gran medida al aumentar la probabilidad de experimentar un curso progresivo y al acortar la latencia hasta la progresión ^29^. Además, también los autores sugieren que, la estratificación por edad, hasta ahora muy poco utilizada puede ser ventajosa, especialmente si el resultado primario es la aparición de esclerosis múltiple secundaria progresiva ^29^. Las etapas tempranas de la enfermedad, especialmente durante edades tempranas, representan una ventana de oportunidad para futuros tratamientos que deben enfocarse en prevenir o retrasar el inicio de la progresión secundaria, el principal determinante del desarrollo de discapacidad permanente.

Tanto en el modelo de regresión simple, como en el modelo de regresión multivariada encontramos que el fenotipo RR no era un factor de influencia de la progresión de la discapacidad de la EM. Stewart *et al.* realizaron un estudio de seguimiento prospectivo en 136 462 pacientes con fenotipo RR y encontraron que una tasa de recurrencia más alta estaba relacionada con una mayor acumulación de discapacidad ^30^ . Estudios sugieren que el fenotipo PP presagia un peor pronóstico que el fenotipo RR o fenotipo SP en términos de discapacidad. Sin embargo, el desarrollo de un curso progresivo de la enfermedad y la tasa de acumulación de discapacidad posterior a la progresión parecen depender de la edad y no se correlacionan con la tasa de acumulación de discapacidad previa a la progresión ^29^. Estos estudios que describen que pacientes con fenotipo PP tienen mayores posibilidades de progresar en discapacidad, respalda a los presentados en este estudio, en donde más del 30% de los pacientes tienen este fenotipo.

Por tratarse de un estudio con seguimiento de una cohorte de pacientes con EM, se produjeron pérdidas durante el seguimiento. Para controlar este sesgo se tuvo en cuenta que cada paciente aportó tiempo mientras que estuvo en el seguimiento, garantizando obtener la información más precisa. En el análisis se incluyó el seguimiento a 167 personas por al menos 20 meses, equivalente al 77,3% de la población de estudio. Sin embargo, a los 40 meses se había perdido alrededor del 50% de la población, esto limita la identificación del momento de la progresión de la discapacidad en este grupo de personas. Asimismo, el muestreo fue no probabilístico, por lo tanto, los resultados no pueden generalizarse al departamento de Antioquia. Al momento del ingreso de los pacientes al estudio, no se encontraban en el mismo estadio de la enfermedad, es decir tenían diferentes niveles de discapacidad, además hubo pérdidas de seguimiento, aunque se tuvieron en cuenta los datos durante el periodo de seguimiento disponible en la historia clínica. No obstante, esto se hizo en el marco de la entrevista clínica con el neurólogo, con quien se revisaron todas las consultas reportadas.

En conclusión, la progresión de la discapacidad en pacientes con EM se ve afectada por muchos factores, y no hay un solo factor independiente. La mediana de tiempo de progresión fue de 72 meses. Las lesiones activas captadas en resonancia magnética y sexo masculino se asociaron con mayor progresión de la discapacidad, con resultados estadísticamente significativos en el modelo multivariado. Por lo tanto, la correcta y oportuna comprensión de los factores de riesgo asociados con la progresión de la discapacidad puede ayudar tanto a asesorar a los pacientes como a mejorar el abordaje y brindar recomendaciones de tratamiento basadas en la evidencia, lo que se traduce a mejorar el pronóstico y la calidad de vida de los pacientes con la patología.
